# Supplementary material for: Screening for anxiety in patients with cancer: Diagnostic accuracy of GAD-7 items considering lowered GAD-7 cut-offs
Source: PLoS One. 2025 Jan 14;20(1):e0316853. doi: 10.1371/journal.pone.0316853 (PMC11731751; doi:10.1371/journal.pone.0316853)
Supplement: Attachment — Submitted filename: Grapp_ GAD7 items cancer _ Response to Reviewers.docx [file pone.0316853.s001.docx]

**Response to Reviewers**

Heidelberg, 21.11.2024

Dear Reviewers,

Thank you very much for your thorough and insightful comments, which have significantly helped us improve our work. Based on your comments, we made substantial revisions, particularly to the Discussion, but also in the Methods section and the Introduction, to enhance clarity and rigor. Please find below our responses to the individual points raised by you in your comments.

During the revision process, we also identified and corrected an error in Table 2 (“Demographic data, clinical data and prevalence of GAD depending on different GAD-7 cut-offs”): the standard deviation previously reported as 9.21 has been corrected to 6.21.

We believe these revisions have significantly strengthened the manuscript and hope it is now suitable for acceptance by PLOS ONE.

Thank you again for your time and consideration. We look forward to your feedback.

Kind regards,

Miriam Grapp (on behalf of all authors)

**Answers to Academic Editor / Journal requirements:**

*1. Please ensure that your manuscript meets PLOS ONE's style requirements, including those for file naming.*

Answer:

We have checked that our manuscript complies with the PLOS ONE style guide and revised the formatting of the level 1 and level 2 headings, the citation of figures in the text, the file names for Fig1 accordingly.

*2. We note that you have indicated that there are restrictions to data sharing for this study. For studies involving human research participant data or other sensitive data, we encourage authors to share de-identified or anonymized data. However, when data cannot be publicly shared for ethical reasons, we allow authors to make their data sets available upon request.*

Answer:

Thank you for this important remark. After consultation with the ethics committee of the University of Heidelberg, we have uploaded a minimal anonymized data set necessary to replicate your study findings on figshare.com.

We have updated our Data Availability statement in the submission form as follows:

“The dataset generated for this study can be found in the Figshare data repository: <https://figshare.com/articles/dataset/Screening_for_anxiety_in_patients_with_cancer_diagnostic_accuracy_of_GAD-7_items_considering_lowered_GAD-7_cut-offs/27880509/1?file=50690409> (<https://doi.org/10.6084/m9.figshare.27880509.v1>)”

**Answers to Reviewer #1**

*1. Expand on Methodological Limitations: While the study mentions the lack of validation with another diagnostic measure for anxiety disorders, this limitation could be explored further. Clarifying how the absence of a clinical interview or additional questionnaires might affect the accuracy of the GAD-7 would strengthen the discussion of limitations and transparency.*

Answer:

Thank you for mentioning this important issue. We agree with the reviewer's comment and are aware that the lack of an objective criterion for validating GAD-7 scores is a major limitation of our study. We have taken this comment very seriously and have expanded on this aspect in the discussion section (see “Limitations”) as follows:

“Structured or semi-structured interviews are considered the gold standard for diagnosing anxiety disorders. However, this was not possible in our study, which focused on a large data set. Data were sampled in the context of routine care, where interviews would be too time consuming. It is important to understand that the GAD-7 is simply a screening tool to identify generalized anxiety disorder, but cannot replace a clinical diagnosis. […]

However, the absence of an additional criterion for evaluating the GAD-7, e.g., HADS as a second screening tool or an interview, results in the criterion standard for this study being the GAD-7 score itself. Thus, the predictor and criterion are not completely independent of each other, which potentially bears the risk that sensitivity and specificity will be overestimated or biased. Therefore, sensitivity and specificity analyses should be interpreted with caution. Further research should therefore incorporate clinical interviews with a sufficiently representative subsample of cancer patients ensure consistency with the GAD-7 results.”

*2. Consider Including TNM Classification: The study could greatly benefit from incorporating the TNM classification of cancer severity. This would allow for a more detailed analysis of how anxiety levels might correlate with the stage of cancer, offering deeper insights into the physical and psychological interaction.*

We completely agree with the reviewer that the study would undoubtedly benefit from incorporating the TNM classification. However, it should be noted that the results are based on a routine questionnaire used in clinical care. This questionnaire is designed to screen for psychological distress, and it also collects some socio-demographic variables. Unfortunately, the TNM classification is not included and is therefore not available to us. We have already mentioned this in the Discussion section (Limitations) and have now expanded the corresponding passage to include the reviewer's suggestions:

“As our data are based on the analysis of a routine questionnaire used in clinical care that focuses on the assessment of psychological distress, no TNM classification was available in the present dataset. Therefore, it was not possible to directly correlate GAD-7 scores with physical disease severity or to perform subgroup analyses. Information on TNM status would allow a more detailed analysis of the possible correlation between anxiety and the cancer stage, providing a more comprehensive understanding of the relationship between physical and psychological symptom dimensions.”

Furthermore, we have added the following passage in the conclusion section to highlight the importance of this aspect for future research:

“It would be beneficial for future research to consider the stage of the disease, as indicated by the TNM classification. Incorporating the TNM classification into analyses could enhance future research by clarifying the relationship between anxiety symptoms and cancer stage, and by deepening insights into the interplay of physical and psychological symptoms.”

*3. Elaborate on the Clinical Implications: The conclusion suggests that a lowered GAD-7 cut-off is beneficial for oncological settings. It would be helpful to include more practical guidelines for clinicians on how this change might influence decision-making in clinical practice, particularly regarding referrals to psychosocial support services.*

Thank you for highlighting this important aspect. We have now added the following section to the Discussion section (under “Clinical implications”):

“Lowering the GAD-7 cut-off would lead to earlier identification of anxiety symptoms in cancer patients, which is crucial given their vulnerability to psychological distress. The main aim of a lowered GAD-7 cut-off is to miss as few patients as possible with a potential anxiety disorder and to give as many as possible access to psycho-oncological interventions. This would imply an increase in the number of patients flagged for potential anxiety disorders. These patients should be referred to the psycho-oncological service at an early stage in order to verify the diagnosis of an anxiety disorder in a two-stage process (preferably with a clinical interview).”

*4. Clarify the Role of Somatic-Emotional and Cognitive Items: The differentiation between somatic-emotional and cognitive items is one of the unique strengths of the study. However, the rationale behind this division could be more fully explained…particularly how this distinction is relevant to the cancer population compared to other populations with anxiety disorders.*

We very much appreciate your positive feedback, in which you mention the distinction between somatic-emotional and cognitive aspects as one of the unique strengths of the study. Furthermore, we acknowledge that the rationale for this division is not elaborated in sufficient detail in our manuscript. Therefore, we have added the following paragraph to the Introduction to make the reasons for the differentiation between somatic-emotional and cognitive items more comprehensible to the reader:

„The GAD-7 also includes items that might be assigned to a more physical (i.e. somatic) symptom dimension (e.g. ‘trouble relaxing’) and items that might be assigned mainly to a cognitive symptom dimension (e.g. ‘not being able to stop/control worrying’). However, there is no such clear and explicit distinction for GAD-7 items as for example in depression screening with PHQ-9 [24] and a discussion about the relevance of somatic items in the identification of anxiety disorders in cancer patients has not yet emerged. Therefore, one aim of the present study is to find out whether a distinction between a somatic-emotional and a cognitive symptom dimension could be relevant in the screening of generalized anxiety disorder in patients with cancer.”

Furthermore, we have expanded on the Methods section and described in more detail our classification process.

“To investigate whether a distinction between a somatic-emotional and a cognitive symptom dimension might be relevant in screening for generalized anxiety disorder in cancer patients, we needed to classify the GAD-7 items into these two dimensions. To the best of our knowledge, such a categorization did not exist before and therefore there was no preliminary work that we could refer to. We therefore used expert consensus for the categorization: seven raters (clinical/scientific experts; psycho-oncologists, physicians) independently assigned each item to one the two symptom dimensions. The classification was determined by the level of agreement between the raters, with items assigned by majority vote when full consensus was not achieved. The resulting categorization used for our analysis is shown in Table 1.”

*5. Strengthen Data Availability Statement: Although the study provides a link to a Figshare repository for data sharing, it's important to ensure that the repository is fully accessible and includes detailed instructions for others who may want to replicate or further explore the analysis. A clear description of what data is available and how it can be accessed is crucial for PLOS ONE's emphasis on open science*.

Thank you for this important information. The editor also pointed out the weaknesses in our Data Availability Statement. After consultation with the Ethics Committee of the University of Heidelberg, we have uploaded an anonymized dataset to figshare.com. Thus, the data set we used for our study is now fully accessible. Furthermore, we have revised the labeling of each variable in the dataset so that the use of the variables is clearly understandable for others who may want to replicate or further explore the analysis.

We have updated our Data Availability statement in the submission form as follows:

“The dataset generated for this study can be found in the Figshare data repository: <https://figshare.com/articles/dataset/Screening_for_anxiety_in_patients_with_cancer_diagnostic_accuracy_of_GAD-7_items_considering_lowered_GAD-7_cut-offs/27880509/1?file=50690409> (<https://doi.org/10.6084/m9.figshare.27880509.v1>)”

*6. Consider Exploring Gender Differences further: The study briefly mentions that the sample is predominantly female. Given that anxiety presentations might differ by gender, a deeper analysis of how GAD-7 scores differ between male and female cancer patients could add an important dimension to the findings.*

Thank you for this valuable comment. Based on the reviewer's comments, we realized that this paragraph needs to be elaborated on in the Discussion section, both to explain why we did not focus on it in this study and to ensure that the relevance of the issue is not overlooked. Moreover, we consider the reviewer's recommendation a great suggestion for future research and have therefore included the following this aspect in the Conclusion section.

Based on this reviewer's comment, we made the following additions to the manuscript:

(1) Discussion/Limitations:

“Moreover, we did not analyze and interpret our results on a sex or gender-specific basis although there is considerable evidence that there are gender differences in the presentation of anxiety symptoms in cancer patients. In particular, recent studies show that female patients are more anxious than male patients across all tumor entities [38]. Given the complexity of our research question, we chose to limit our scope. We did not address gender differences in anxiety disorders and in the presentation of anxiety symptoms. This could slightly restrict the generalizability of our findings.”

(2) Conclusion:

“Another important aspect for further research is a deeper analysis of how GAD-7 scores and the extent of different anxiety symptoms differ between male and female patients with cancer. Given the known gender differences regarding the suffering of anxiety symptoms in oncological patients, this could add an important dimension to our findings.”

**Answers to Reviewer #2**

*The manuscript by Grapp et al. is an original work that examines the diagnostic accuracy of the items of the GAD-7 scale and, based on this, draws conclusions about the ideal cut-off value of the GAD-7 in cancer patients. It is therefore a clinically relevant and interesting work. The manuscript, which is well written in terms of style, comes from a renowned group in the field of psycho-oncology. Although the manuscript is important, it raises some questions that the authors might consider in the context of a revision:*

Answer:

Thank you very much for your encouraging feedback on our manuscript. We are grateful for your recognition of the clinical relevance and importance of our work, as well as your kind words regarding its style and our contributions to the field of psycho-oncology.

*1. Although GAD 7 is named after generalised anxiety disorder, it is also used to screen for other anxiety disorders. In the manuscript, the authors only refer to the accuracy with regard to generalised anxiety disorder. At the very least, the method section should include a justification for not also examining the other anxiety disorders. If necessary, this should be mentioned as a limitation in the discussion.*

Answer:

Thank you for this important comment. We have now provided a more detailed explanation in the Methods section and believe it may not be necessary to further discuss this issue in the Limitations section of the Discussion.

We added the following paragraph to the Methods section:

“Although initially developed as a screening tool for Generalized Anxiety Disorder, the GAD-7 also demonstrates acceptable sensitivity and specificity for identifying other anxiety disorders (Panic Disorder, Social Anxiety, and PTSD) [15]. In the present study, however, the focus was specifically on the symptoms of generalized anxiety disorder. Consequently, the diagnostic accuracy of the GAD-7 was examined solely in relation to generalized anxiety disorder, and other anxiety disorders were not considered.”

*2. While a clear distinction is made in the diagnosis of depressive disorders between somatic and non-somatic items (e.g. somatic items of the PHQ-9: ‘feeling tired or having little energy’ or ‘poor appetite or overeating’), this distinction appears not really comprehensible for the GAD-7. It is not clear to me why the items on nervousness, difficulty relaxing, restlessness, irritability and anxiety are emphasised with regard to their somatic component, in contrast to the corresponding items of the PHQ-9. This subdivision of the GAD-7 seems somewhat arbitrary to me. The authors are asked to reconsider their argumentation and to check whether this separation is really necessary.*

Answer:

We understand your concerns at this point. To the best of our knowledge, this separation has not been addressed in previous studies. As such, the categorization of the items into somatic-emotional and cognitive aspects—based on the approach used for the PHQ-9 items—represents one of the innovative aspects of our study. However, we agree that this categorization can only be considered as a model.

Based on the feedback from both reviewers, we have also realized that we need to better explain the rationale behind this categorization, its process, and the potential limitations. As a result, we have revised the manuscript in several places to…

1. clarify the rationale for dividing the items into somatic-emotional vs. cognitive categories (Introduction),
2. make the classification process more transparent (Methods),
3. highlight the limitations and the model-like nature of this categorization (Discussion).

The following passage was added to the Introduction:

“[Another important aspect is that in some studies on depressive disorders in patients with cancer, a symptom overlap with somatic disease is reported [17-19], and the question of whether somatic items are appropriate for screening depressive disorders has been the subject of controversy [20-23]]. The GAD-7 also includes items that might be assigned to a more physical (i.e. somatic) symptom dimension (e.g. ‘trouble relaxing’) and items that might be assigned mainly to a cognitive symptom dimension (e.g. ‘not being able to stop/control worrying’). However, there is no such clear and explicit distinction for GAD-7 items as for example in depression screening with PHQ-9 [24] and a discussion about the relevance of somatic items in the identification of anxiety disorders in cancer patients has not yet emerged. Therefore, one aim of the present study is to find out whether a distinction between a somatic-emotional and a cognitive symptom dimension could be relevant in the screening of generalized anxiety disorder in patients with cancer.”

Furthermore, we have expanded on the Methods section and described in more detail our classification process:

“To investigate whether a distinction between a somatic-emotional and a cognitive symptom dimension might be relevant in screening for generalized anxiety disorder in cancer patients, we needed to classify the GAD-7 items into these two dimensions. To the best of our knowledge, such a categorization did not exist before. We therefore used expert consensus for the categorization: seven raters (clinical/scientific experts; psycho-oncologists, physicians) independently assigned each item to one the two symptom dimensions. The classification was determined by the level of agreement between the raters, with items assigned by majority vote when full consensus was not achieved. The resulting categorization used for our analysis is shown in Table 1.”

In the Discussion section (under Limitations), the following paragraph has been added:

“Some results and conclusions of this paper are based on the categorization of the GAD-7 items into somatic-emotional and cognitive items. As already mentioned in the methods section, such a classification has not yet been made, which means that there is no preliminary work or publications that we could have referred to. Instead, the classification was based on an expert consensus and is to be regarded as a model. The aim of this model was to investigate whether a division into somatic-emotional and cognitive items – in analogy to the extensive discussion regarding the PHQ-9 items – could also be relevant for patients with cancer.”

*3. As the authors correctly note in the discussion, a criterion-standard instrument for generalized anxiety is missing. The criterion standard in this study is the GAD-7 score itself, so that the items are correlated with themselves as part of the GAD-7 score when determining diagnostic accuracy. An external and thus independent criterion standard is missing. The authors might argue why they believe that evidence of the cut-off value of the GAD-7 can be derived from this partial self-correlation, for which external criterion standards are usually used. I think, the rationale for this should be presented in a differentiated manner in the methods section and critically discussed in the discussion section.*

Answer:

Thank you for raising this important issue. We fully acknowledge that the lack of an objective criterion to validate the GAD-7 values is a major limitation of our study. We appreciate your helpful suggestions on how to address this limitation in more detail. In response, we have expanded the explanation of the reasons for the absence of an external criterion standard in the Methods section and have discussed this limitation more critically in the Discussion section.

We have added the following passages to the Methods section (under ‘Measures and criterion references’):

“As the data were collected as part of a routine screening process, there is no external, and therefore independent, criterion that can be used to validate the GAD-7 score. It is therefore not possible to completely eliminate the possibility of partial self-correlation with GAD-7 items and the GAD-7 score. Therefore, the risk that the diagnostic accuracy measures used in this study may be overestimated or biased must be considered when analyzing and interpreting the results.”

In the Discussion section, we have added the following passages to critically discuss the limitations due to the lack of an external criterion and to provide more transparency regarding this methodological limitation.

“Structured or semi-structured interviews are considered the gold standard for diagnosing anxiety disorders. However, this was not possible in our study, which focused on a large data set. Data were sampled in the context of routine care, where interviews would be too time consuming. It is important to understand that the GAD-7 is simply a screening tool to identify generalized anxiety disorder, but cannot replace a clinical diagnosis. […]

However, the absence of an additional criterion for evaluating the GAD-7, e.g., HADS as a second screening tool or an interview, results in the criterion standard for this study being the GAD-7 score itself. Thus, the predictor and criterion are not completely independent of each other, which potentially bears the risk that sensitivity and specificity will be overestimated or biased. Therefore, sensitivity and specificity analyses should be interpreted with caution. Further research should therefore incorporate clinical interviews with a sufficiently representative subsample of cancer patients ensure consistency with the GAD-7 results.”

*4. It is not clear to me why the diagnostic accuracy of the individual items of the GAD-7 is calculated. After all, the aim of clinical application is to ensure the accuracy of the entire GAD-7 scale or the GAD-2 short form, not the accuracy of individual items. The fact that a scale consists of items of varying difficulty is actually desirable in scales! In my opinion, if the validity of parts of the GAD-7 is to be examined, then it should be the validated short form of the GAD-7, not in the individual items. At the very least, the authors might provide a better explanation of the rationale for this approach.*

Answer:

We greatly appreciate your feedback. Our manuscript does not focus on the quality criteria of the GAD-7 or GAD-2 questionnaires, which, as you correctly noted, depend on a balanced composition of items. Instead, we focus on the symptom level—specifically, the symptoms of generalized anxiety disorder (GAD)—as captured by the individual items of the questionnaire. The rationale behind this approach is to investigate whether, analogous to depression, an overlap exists between anxiety symptoms and symptoms related to cancer or cancer treatment in cancer patients. Since some anxiety symptoms are also observed in individuals with cancer who do not meet the criteria for generalized anxiety disorder, it is crucial to examine the diagnostic value of each individual symptom. This approach also enables us to explore whether the conventional symptoms of generalized anxiety disorder lack specificity in the oncological context.

To improve transparency and better clarify our approach, we have added the following text to the Introduction section. (The issue of symptom overlap is addressed in the preceding section of the Introduction, as noted in the previous reviewer’s comments.)

“Thus, the objective of our study is not to evaluate the quality criteria of the GAD-7 questionnaire itself; rather, our focus is on the individual symptoms of generalized anxiety disorder as assessed by the GAD-7 items. Given that some anxiety symptoms are also present in individuals with cancer who do not have generalized anxiety disorder, this analysis provides an opportunity to examine whether the conventional symptoms of generalized anxiety disorder may lack diagnostic specificity in an oncological context. Additionally, we explore whether diagnostic accuracy might be enhanced by adjusting the GAD-7 cut-off.”

*5. In the methods section, a brief explanation should be given as to why the data are not very recent (2018); in the discussion, it could be discussed whether this could have an impact on the results.*

Answer:

Thank you for highlighting this aspect. We have included an explanation in the Methods section as to why the data collected may be not very recent and added possible implications of this for the results in the Discussion. The amended text passages are as follows:

Methods:

“Routine data was collected as part of a standardized bio-psycho-social distress screening between 2011 and 2018, as this period was characterized by a consistent screening methodology and standardized data collection protocols. After 2018, changes in the screening process, modifications in the variables collected, and a prolonged interruption of screening activities due to the COVID-19 pandemic introduced significant variations. Therefore, the selected timeframe ensures the comparability and reliability of the data.”

Discussion/Limitations:

“[It should also be noted that our data was collected between 2011 and 2018 and therefore originates from the period before the COVID-19 pandemic]. The COVID-19 pandemic may have introduced new anxiety triggers and altered baseline anxiety levels in recent cancer patients due to increased health-related uncertainties and potential disruptions in social support systems [36]. The data in this study do not account for these pandemic-related influences on the prevalence and significance of anxiety symptoms, which may lead to differences in anxiety profiles between current patients and those in the 2011-2018 dataset, potentially affecting the comparability of findings. Additionally, shifts in mental health awareness and decreasing stigma surrounding anxiety may have influenced self-reporting practices over time. Patients today may feel more comfortable disclosing symptoms than those in earlier years, possibly leading to a slight underreporting of anxiety prevalence in the 2011-2018 dataset.”

*6. Results section (3.1): The GAD-7 is not used to measure prevalences! It only measures the frequency of screening diagnoses!*

Answer:

Thank you very much for this important hint. We have changed the respective sentence in the Results section as follows:

“The frequencies and mean scores of the GAD symptoms (represented by the individual GAD-7 items) depending on different GAD-7 cut-offs as well as for the total sample are provided in Table 3.”

*7. There are high frequencies for anxiety disorders at the cut-off scores of 7 and 8 (22 and 28%). Are these high prevalences clinically plausible? In my opinion, this should be discussed.*

Answer:

Thank you for this comment, which we are pleased to address. We have further developed this aspect, which we had previously mentioned in the discussion under “Main findings” and revised the previous text as follows:

“Our results indicate that, among oncology patients, the optimal balance between sensitivity, specificity, PPV, and NPV of the GAD-7 is achieved with a cut-off score of ≥ 8. This aligns with findings from Plummer et al.’s meta-analysis [15] and Esser et al.’s study [16]. Lowering the GAD-7 cut-off in our study significantly increased the percentage of patients who screened positive for generalized anxiety disorder, with rates reaching 28.2% at a cut-off of ≥ 7 and 22.3% at ≥ 8. We consider these elevated rates to be clinically plausible, given the unique characteristics of our target group, consisting of cancer patients recently diagnosed and at the beginning of their treatment. According to existing literature, anxiety in patients with cancer follows distinct trajectories from diagnosis through the end of first-line treatment to long-term follow-up. Anxiety is particularly prominent in the early stages of diagnosis and at the onset of acute treatment, often reflecting a reaction to the diagnosis and anticipated treatment [4, 37].”

*8. Tab. 3 and Tab. 4: Here I would recommend writing out the items – this makes it easier for the reader who does not know the individual items by heart to find his way around.*

Answer:

Thank you for this valuable recommendation, which we are pleased to follow. We have now written out the items in Tables 3 and 4.

*9. Conclusions: The conclusion that different response patterns were found for the somatic-emotional and cognitive items is not valid, as these differences have not been statistically tested.*

Answer:

Thank you for this important and critical comment. We have rephrased the relevant sentence in the Conclusion section to be more tentative and have added a sentence acknowledging that this observation has not been statistically tested and should therefore be considered with caution.

Conclusion:

“Furthermore, we observed differing trends between the somatic-emotional and the cognitive GAD-7 items. While the cognitive items appear to be more relevant for identifying severe GAD with higher cut-off values, the somatic-emotional items seem to be more relevant for identifying low to moderate GAD. However, it is important to note that these trends have not been statistically tested, and thus any conclusions regarding their significance should be considered tentative.”
